# A high-throughput real-time PCR tissue-of-origin test to distinguish blood from lymphoblastoid cell line DNA for (epi)genomic studies

**DOI:** 10.1038/s41598-022-08663-6

**Published:** 2022-03-18

**Authors:** Lise M. Hardy, Yosra Bouyacoub, Antoine Daunay, Mourad Sahbatou, Laura G. Baudrin, Laetitia Gressin, Mathilde Touvier, Hélène Blanché, Jean-François Deleuze, Alexandre How-Kit

**Affiliations:** 1 Laboratory for Genomics, Foundation Jean Dausset-CEPH, 75010 Paris, France; 2 Laboratory of Excellence GenMed, Paris, France; 3 Laboratory for Human Genetics, Foundation Jean Dausset-CEPH, Paris, France; 4 Centre de Ressources Biologiques, CEPH Biobank, Foundation Jean Dausset-CEPH, Paris, France; 5 Sorbonne Paris Nord University, Nutritional Epidemiology Research Team (EREN), Epidemiology and Statistics Research Center Inserm U1153, Inrae U1125, Cnam, University of Paris (CRESS), Bobigny, France; 6 Centre National de Recherche en Génomique Humaine, CEA, Institut François Jacob, Evry, France; 7 Laboratory for Sciences of Biobanking, Foundation Jean Dausset-CEPH, Paris, France

**Keywords:** Genetic markers, DNA and RNA, Methylation analysis, High-throughput screening, PCR-based techniques, DNA methylation

## Abstract

Lymphoblastoid cell lines (LCLs) derive from blood infected in vitro by Epstein–Barr virus and were used in several genetic, transcriptomic and epigenomic studies. Although few changes were shown between LCL and blood genotypes (SNPs) validating their use in genetics, more were highlighted for other genomic features and/or in their transcriptome and epigenome. This could render them less appropriate for these studies, notably when blood DNA could still be available. Here we developed a simple, high-throughput and cost-effective real-time PCR approach allowing to distinguish blood from LCL DNA samples based on the presence of EBV relative load and rearranged T-cell receptors γ and β. Our approach was able to achieve 98.5% sensitivity and 100% specificity on DNA of known origin (458 blood and 316 LCL DNA). It was further applied to 1957 DNA samples from the CEPH Aging cohort comprising DNA of uncertain origin, identifying 784 blood and 1016 LCL DNA. A subset of these DNA was further analyzed with an epigenetic clock indicating that DNA extracted from blood should be preferred to LCL for DNA methylation-based age prediction analysis. Our approach could thereby be a powerful tool to ascertain the origin of DNA in old collections prior to (epi)genomic studies.

## Introduction

Lymphoblastoid cell lines (LCLs) result from the immortalization of B-lymphocytes from blood samples though stable infection with Epstein–Barr Virus (EBV) of the herpesvirus family in vitro^1–3^. EBV infection is mediated through the interaction of viral gp350 and gp42 glycoproteins with B-lymphocytes CD21/CR2 and HLAII receptor proteins, while the viral genome is maintained and replicated in the cells as episomal DNA or integrated in the nuclear genome in a lesser proportion^4,5^. The transformation of B-cells into proliferating and immortalized LCLs is under the control of latency III viral gene expression program comprising more than ten coding (*EBNA*s and *LMP*s) and non-coding (*EBER*s, *miR-BHRF1*s and *miR-BART*s) genes^5,6^.

Since the establishment of first LCLs, these cells have proven to be extremely useful in several genetic, functional and pharmacogenomic studies as well as for the development of immunotherapies^7–10^. LCLs allow access to unlimited amount of DNA and overcome the need of high amount of blood and/or resampling from donors, while allowing their conservation in DNA and cell line biobanks (e.g. CEPH Biobank, https://cephb.fr/, Coriell Biobank, https://www.coriell.org/1/Browse/Biobanks) and distribution to the scientific and biomedical community^7^.

Historically, DNAs from LCLs have allowed to set up some worldwide-used reference DNA samples such as those from the CEPH reference families^11^ or the HGDP-CEPH (Human genome Diversity Project-Centre d’Etude du Polymorphisme Humain)^12^ panels, which have extensively been used in several large scale genetic studies, including the construction of human genetic maps^11^, description and analysis of genetic variations across human populations (HGDP-CEPH, HAPMAP and 1000 genomes)^13–15^, and genome-wide association studies^16^. In addition to genetic studies, LCLs have also been used as a surrogate biological material that could be representative of blood in other genomic^17,18^, transcriptomic^19–21^ and epigenomic^22,23^ studies.

However, several comparative studies highlighted the presence of modifications in the (epi)genome and transcriptome of LCLs compared to blood due to immortalization and in vitro culture, as well as the absence of representativity of all types of blood cells. These modifications included few mutations^24–26^, some copy number variations and chromosomal aberrations^1,27,28^, mtDNA mutations and copy number changes^28–30^, frequent DNA methylation variations^31–34^ as well as modification of transcriptomes^35–38^. As a result LCLs may not completely reflect the tissue of origin and most of these studies have recommended their use with caution in genomic and transcriptomic studies and even more in epigenomic studies^28,31–35,38,39^. Thus, the use of blood should be preferred to LCLs for these types of studies, notably when blood DNA or RNA samples could still be available.

In this context, our study aimed to develop a simple and efficient high-throughput real-time PCR approach allowing the rapid identification of the biological material from which the DNA was extracted (blood or LCL). The method is intended to be used on large scale DNA collections as a screening and/or quality control test that could be used to validate, ascertain or identify their tissue of origin i.e. blood or LCL, prior to downstream (epi)genomic studies. The approach is based on the detection of different genetic features specific either to LCLs or blood DNA, including the relative quantification of *EBV* genome whose copy number is very high in LCLs and the detection of rearranged *TCR*_*β*_ and *TCR*_*γ*_ that are specific to T-cells in blood. It was developed and optimized using 458 blood samples from healthy donors from the SU.VI.MAX cohort^40^ and the French blood bank (EFS) as well as 316 LCL reference DNA samples from CEPH families^11^.

We further applied our tissue-of-origin test on 1957 DNA samples from the CEPH Aging cohort, which was recruited during the years 1990 to 2000 and comprises more than 2000 nonagenarians, centenarians and super-centenarians as well as their offspring^41,42^. The collection includes more than 10,000 DNA samples extracted from blood or LCLs, but this information was dated, uncertain or sometimes missing and needed to be verified or determined. Following the identification of their tissue of origin, we performed DNA methylation-based age prediction on a subset of DNA samples from blood and LCLs using an epigenetic clock based on three loci and pyrosequencing^43,44^ and compared the age predictions to their chronological ages. The results confirmed that the use of blood DNA should be preferred over LCL DNA for DNA methylation analyses and that the developed tissue of origin test could be a useful tool for the rapid identification, verification or validation of the DNA origin. It could be easily implemented in biobanks and used along with the other quality controls of DNA on several large scale and/or ancient DNA collections prior to (epi)genomic studies.

## Materials and methods

### Ethics statement

The study was conducted in accordance with current ethical and legal frameworks and approved by an institutional review board (comité consultatif de protection des personnes dans la recherche biomedicale, CCPPRB Paris-Saint-Antoine, approval No. 00479). Informed consents were obtained from all participants.

### Reference blood and lymphoblastoid cell line DNA

DNA extracted from LCL and blood was used as reference for the development of real-time PCR assays (Table 1), including 316 LCL DNA from CEPH reference families^11^ provided by the CEPH Biobank, 364 blood DNA of healthy individuals from the SU.VI.MAX cohort and 93 blood DNA of healthy donors^43,45^ from the French blood bank, EFS (Etablissement Français du Sang, Paris, France—research agreement 15/EFS/012). Sex and age at collection of the individuals from the different cohorts were given in Table 1.

**Table 1:** Descriptive statistics of the DNA samples used from the four collections used.

Cohort characteristics	CEPH reference families (n = 316)	SU.VI.MAX (n = 364)	EFS (n = 93)	CEPH aging (n = 1813)	
				Nonagenarians and centenarians (NC, n = 1346)	Nonagenarians and centenarians’ offspring (NCO, n = 467)
Tissue-of-origin of DNA	LCL	Blood	Blood	LCL and blood	LCL and blood
Age^a^ in years, M ± SD (range)	48.9 ± 22.1 (18–97)^b^	48.9 ± 5.9 (35–61)	41.6 ± 13.4 (19–69)	99.3 ± 3.8 (90–110^+^)	68.4 ± 9.2 (48–90)
Females, n (%)	158 (50%)	182 (50%)	40 (43%)	1032 (76.7%)	262 (56.1%)

^a^Age at collection.

^b^Known for 214 samples.

### CEPH aging cohort DNA

The CEPH aging cohort comprises 1561 French nonagenarians, centenarians and super-centenarians born between 1875 and 1910 and recruited during the years 1990 to 2000, including 528 individuals from 228 families, as well as 468 of their offspring belonging to 147 families^41,42^. The cohort comprises 10,173 DNA extracted from blood or LCL and the information about their tissue of origin was sometimes uncertain or missing. 1957 DNA samples from 1813 individuals were used for the assessment of their blood or LCL origin.

### DNA quantification and pre-PCR processing

DNA from all collections was quantified using Quant-IT™ dsDNA Broad-Range assay kit on a Synergy HTX (BioTek) for fluorescence measurement and analysis (Centre de Ressources Biologiques, CEPH Biobank, Foundation Jean Dausset—CEPH) or Qubit™ dsDNA BR assay Kit on a Qubit 3 Fluorometer (Thermo Fischer Scientific), according to the manufacturer’s instructions. DNA sample concentrations were equalized to 5 ng/µL and dispensed in 96 wells PCR plates using a JANUS Liquid Handler Workstation (Perkin Elmer).

### Real-time PCR

*EBV*, *GAPDH*, *TCR-β* and *TCR-γ* real-time PCR assay primers were given in Supplementary Table 1. PCR primers and reactions conditions were modified from Sahin et al.^46^ for *EBV*, Sprouse et al.^47^ for *TCR*_*γ*_ and van Dongen et al.^48^ for *TCR*_*β*_*.* All PCR reactions were performed in 384 PCR plates on a LightCycler 480 (Roche) with 10 ng of DNA in 10 µL volume using a Bravo Automation Liquid Handling Platform (Agilent) for plate preparation. The PCR mix included 1 × HotStar Taq DNA polymerase buffer, 1.6 mM of additional MgCl_2_, 200 µM of each dNTP, 1.5 µM of SYTO™ 9 (Invitrogen), 200 nM of each primer and 0.5 U of HotStar Taq DNA polymerase (Qiagen). PCR conditions included an initial denaturation step performed for 10 min at 95 °C, followed by 50 cycles of denaturation, annealing and elongation (Supplementary Table 1). The final step included a melting curve (0.2 °C per acquisition) from 65 to 95 °C. Crossing point (C_t_) values from *GAPDH*, *EBV* and *TCR*_*γ*_ PCR assays as well as the melting temperature(s) (T_m_) of *TCR*_*β*_ amplicons were obtained using the 2nd derivative max analysis and the melting curve analysis modules of the LightCycler^®^ 480 SW 1.5.1 software (Roche), respectively. A C_t_ value of 40 for *EBV* assay and 45 for *TCR*_*γ*_ assay was set for all samples with no PCR amplification to allow analyses.

### DNA methylation analysis and age predictions

One µg of DNA was bisulfite-treated using the EpiTect Bisulfite 96 Kit (Qiagen) according to the manufacturer’s instructions. Bisulfite-converted DNA was quantified using the quantitative real-time PCR QC1 methylight assay^49^ and diluted to a final concentration of 20 ng/µL for PCR. 20 ng of bisulfite-treated DNA was used as template for each PCR reaction using three bisulfite-specific PCR primer pairs (*ELOVL2*, *KLF14* and *TRIM59*) according to the PCR reaction and cycling conditions described in Ref.^43^. After PCR, 10 µL of amplified product was purified and prepared for pyrosequencing using the pyrosequencing primers and assays described in Ref.^43^ and according to the detailed protocol described in Refs.^50,51^. DNA methylation analysis was performed using the PyroMark Gold SQA Q96 Kit (Qiagen) on a PyroMark Q96 MD (Qiagen) and the data were analysed with PyroMark CpG software (Qiagen). DNA methylation-based age predictions were performed using DNA methylation values of *ELOVL2* (CpG_5_), *KLF14* (CpG_2_) and *TRIM59* (CpG_5_) with a multiple linear regression model (predicted age = − 20.372 + 0.830 × *ELOVL2* (CpG_5_) + 1.723 × *KLF14* (CpG_2_) + 0.715 × *TRIM59* (CpG_5_))^43,44^.

### Statistical analysis

*GAPDH* was used as a control single copy gene in genomic DNA for the normalization of C_t_ values. C_t *GAPDH*_/C_t *Gene/Genome of interest*_ ratios were calculated for *EBV* and *TCRγ* and used to classify DNA samples in three different groups (blood, LCL and uncertain origin) according to C_t *GAPDH*_/C_t *Gene/Genome of interest*_ ratio using two thresholds chosen empirically. For *TCR*_*β*_, the highest melting temperature (T_m_) was selected to distinguish between blood and LCL DNA using a single threshold. The sensitivity, specificity, positive predictive value (PPV), negative predictive value (NPV) and accuracy of the three real-time PCR tissue-of-origin tests used alone or in combination were calculated. For each calculation, samples identified as blood were considered as positive results while those identified as LCL and of uncertain origin were considered as negative.

## Results

### Strategies for distinguishing blood from LCL DNA

Our study aimed to develop a real-time PCR approach allowing to differentiate DNA extracted from blood or LCL. We first searched for genetic features specific to LCL or blood DNA. The first genetic feature relied on the detection of EBV genomes in the DNA, whose copy number is high in LCL DNA (2 to 500 copies per diploid genome equivalent)^52^ and low to zero in blood DNA of individuals with no ongoing EBV infection or EBV-associated diseases^53–55^. We also searched for other genetic features that could be specific to blood DNA and absent in LCL DNA and identified rearranged T-cell receptor (TCR) genes and extra-chromosomal signal joint T-cell receptor excision circles (sjTREC) that are specific from T lymphocytes^56,57^. As sjTRECs drastically decrease in blood with age until being barely detectable around 80 years old^58^, we focused on rearranged *TCR* genes from T lymphocytes whose number is maintained throughout life^59,60^. We further restricted our choice to *TCR*_*β*_ and *TCR*_*γ*_ and excluded *TCR*_*δ*_, as it is known to be frequently rearranged in B-lymphocytes^61^, and *TCR*_*α*_ due to the high complexity of this gene locus, which presents a large number of V/J segments, and of its rearrangement^48,62^. Thus, to develop our tissue-of-origin test we decided to focus on three genetic features i.e. EBV DNA relative load and rearranged *TCR*_*β*_ and *TCR*_*γ*_.

### *EBV* real-time PCR assay

**Figure 1:**
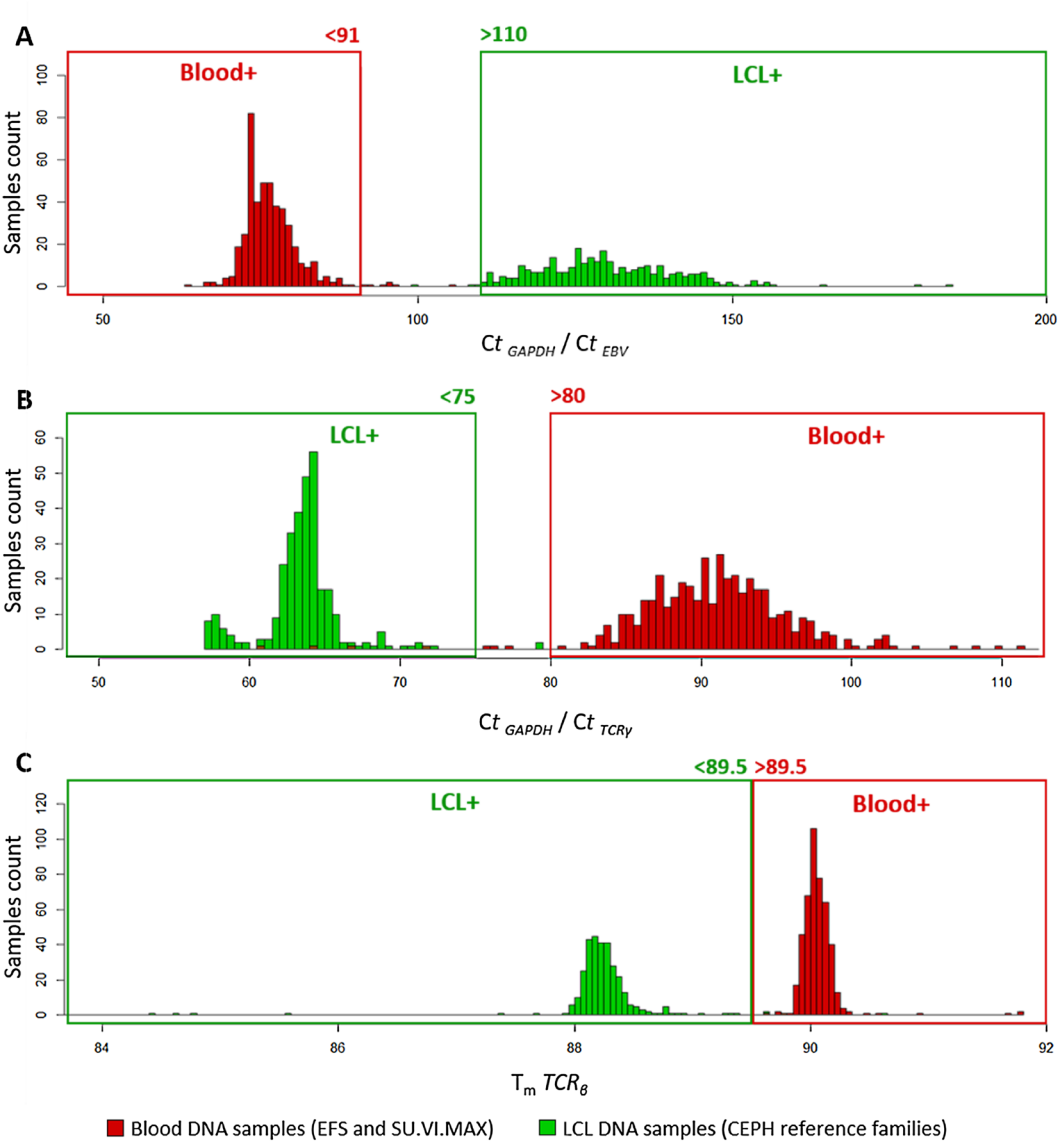
Distribution of C_t *EBV*_/C_t *GAPDH*_ ratios, C_t *TCRγ*_/C_t *GAPDH*_ ratios and mean *TCR*_*β*_ T_m_ from blood DNA from EFS and SU.VI.MAX (n = 457) and LCL DNA from CEPH reference families (n = 316) using real-time PCR assays. (**A**) Distribution of C_t *EBV*_/C_t *GAPDH*_ ratios based on *EBV* and *GAPDH* real-time PCR assays. (**B**) Distribution of C_t *TCRγ*_/C_t *GAPDH*_ ratios based on *TCR*_*γ*_ and *GAPDH* real-time PCR assays. (**C**) Distribution of mean *TCR*_*β*_ T_m_ based on *TCR*_*β*_ real-time PCR assay. The chosen thresholds for each test are given above the frameworks.

For the development of our tissue-of-origin PCR test, we first developed, optimized and evaluated the *EBV* PCR assay using DNA samples of known origin. DNA extracted from blood were obtained from EFS healthy donors (n = 93) and from healthy individuals of the SU.VI.MAX cohort (n = 364) (see “Materials and methods” and Table 1), while DNA extracted from LCLs were from CEPH reference families (n = 316). 10 ng DNA from blood and LCL were used for this assay as well as for all other PCR assays in order to limit the amount of DNA required for each test. We also used a PCR assay targeting *GAPDH* single copy gene as a control to assess the quantification of our DNA samples and to test for the amplifiability of DNA samples. The results showed that its C_t_ values are comparable across all the tested samples (Supplementary Fig. 1) indicating no quantification bias and/or DNA with extreme degradation. Moreover, *GAPDH* assay and C_t_ values were used to normalize the C_t_ value of the *EBV* PCR assay for every tested DNA sample. Figure 1A showed the bimodal distribution of blood and LCL DNA samples according to their C_t *GAPDH*_/C_t *EBV*_ ratio. We decided to set empirically two cut-offs for C_t *GAPDH*_/C_t *EBV*_ ratio, with a first threshold at 91 below which all samples are considered as blood (Fig. 1A). On the contrary, DNA samples origin was considered as LCL when C_t *GAPDH*_/C_t *EBV*_ was higher than 110, which was the second threshold set. Samples whose ratio was comprised between 91 and 110 were classified as samples of uncertain origin. With our set thresholds for C_t *GAPDH*_/C_t *EBV*_ ratio, our EBV tissue-of-origin test presented a strong specificity, sensitivity and accuracy (98.5%, 100% and 0.99, respectively, Table 2). As we aimed to exclude false positive samples that could hinder downstream (epi)genomic analyses if LCL DNA samples were misclassified as blood samples, our *EBV* PCR test resulted in 100% PPV indicating a very high confidence for identification of DNA extracted from blood (Table 2).

**Table 2:** Calculations of sensitivity, specificity, PPV, NPV and accuracy for tissue-of-origin tests.

Tissue-of-origin assay	Sensitivity (%)	Specificity (%)	PPV (%)	NPV (%)	Accuracy
C_t *GAPDH*_/C_t *EBV*_	98.5	100.0	100.0	97.8	0.991
C_t *GAPDH*_/C_t *TCRγ*_	94.3	99.4	99.5	92.4	0.962
T_m_ *TCR*_*β*_	98.2	98.7	99.1	97.5	0.984
Combined *EBV*, *TCR*_*γ*_ and *TCR*_*β*_ tests	98.5	100.0	100.0	97.8	0.991

For our calculations, blood + was considered as the positive result, and LCL + and uncertain origin as negatives results.

### ***TCR***_γ_ real-time PCR assay

Similarly to the *EBV* assay, we developed a second tissue-of-origin real-time PCR assay to distinguish blood from LCL DNA samples based on a second genetic feature that is assumed to be absent in LCL DNA and present in blood DNA, i.e. rearranged *TCR*_*γ*_ genes. The TCR_γ_ assay used one primer pair corresponding to *V*_*II*_ and *J*_*I/II*_ segments and amplifying a large proportion of the recombined *TCR*_*y*_ gene repertoire^47^. Indeed, a small number of *V* and *J* segments allowed the use of a limited number of consensus primers, leading to amplification of a majority of rearranged *TCR*_*γ*_ genes^47,63^. C_t_ values for rearranged *TCR*_*γ*_ assay were normalized with C_t *GAPDH*_ values to obtain a ratio that also presented a bimodal distribution among blood and LCL DNA samples (Fig. 1B). The calculated sensitivity for blood DNA detection was of 94.3% while its specificity was of 99.4% for a PPV of 99.5% and an overall accuracy of 0.96 (Table 2). In comparison, the *TCR*_*γ*_ test thereby presented a slightly lower performance than the *EBV* assay for the identification of the blood origin of DNA (Table 2).

### ***TCR***_***β***_ real-time PCR assay

For our third tissue-of-origin assay, we considered another genetic feature specifically expressed in blood tissue but not in LCLs, i.e. the rearranged *TCR*_*β*_ gene. The *TCR*_*β*_ gene contains many *V/D/J* variable regions, which are rearranged through the maturation of T lymphocytes. Thereby, blood contains a huge diversity of recombined TCR_β_ receptors, which required the use of multiplexed primers for the amplification of a portion of this repertoire. Our selected primers allowed the amplification of *D*_*β1*_ segment rearranged with any *J*_*β1*_–*J*_*β6*_ segments of the *TCR*_*β*_ gene^48^. Due to the use of several PCR primers in a single multiplexed PCR reaction that generated primer dimers as well as non-specific amplifications, C_t_ values from blood and LCL DNA samples were close and did not allow the use of a C_t *GAPDH*_/C_t *TCRβ*_ ratio for this test to distinguish blood from LCL DNA (Supplementary Fig. 1 and 2). Thus, we chose to look at the melting temperature values (T_m_) obtained with melting curve analysis after PCR amplification: T_m_ results for blood DNA samples were over 89.5 °C with a low proportion of primer dimers with lower T_m_ (< 89.5 °C), whereas LCL DNA melting curves presented only T_m_ values under 89.5 °C corresponding to primer dimers and non-specific amplification products (Supplementary Fig. 2). When we used the highest T_m_ obtained for *TCR*_*β*_ amplicons, we obtained a bimodal distribution in blood and LCL DNA samples allowing to distinguish them (Fig. 1C). We used a threshold of 89.5 °C that allowed to identify blood DNA samples with 98.2% sensitivity, 98.7% specificity, 99.1% PPV and 0.98 accuracy (Fig. 1C, Table 2).

### Combination of the three tissue-of-origin PCR tests strongly excluded false positive blood DNA samples

**Figure 2:**
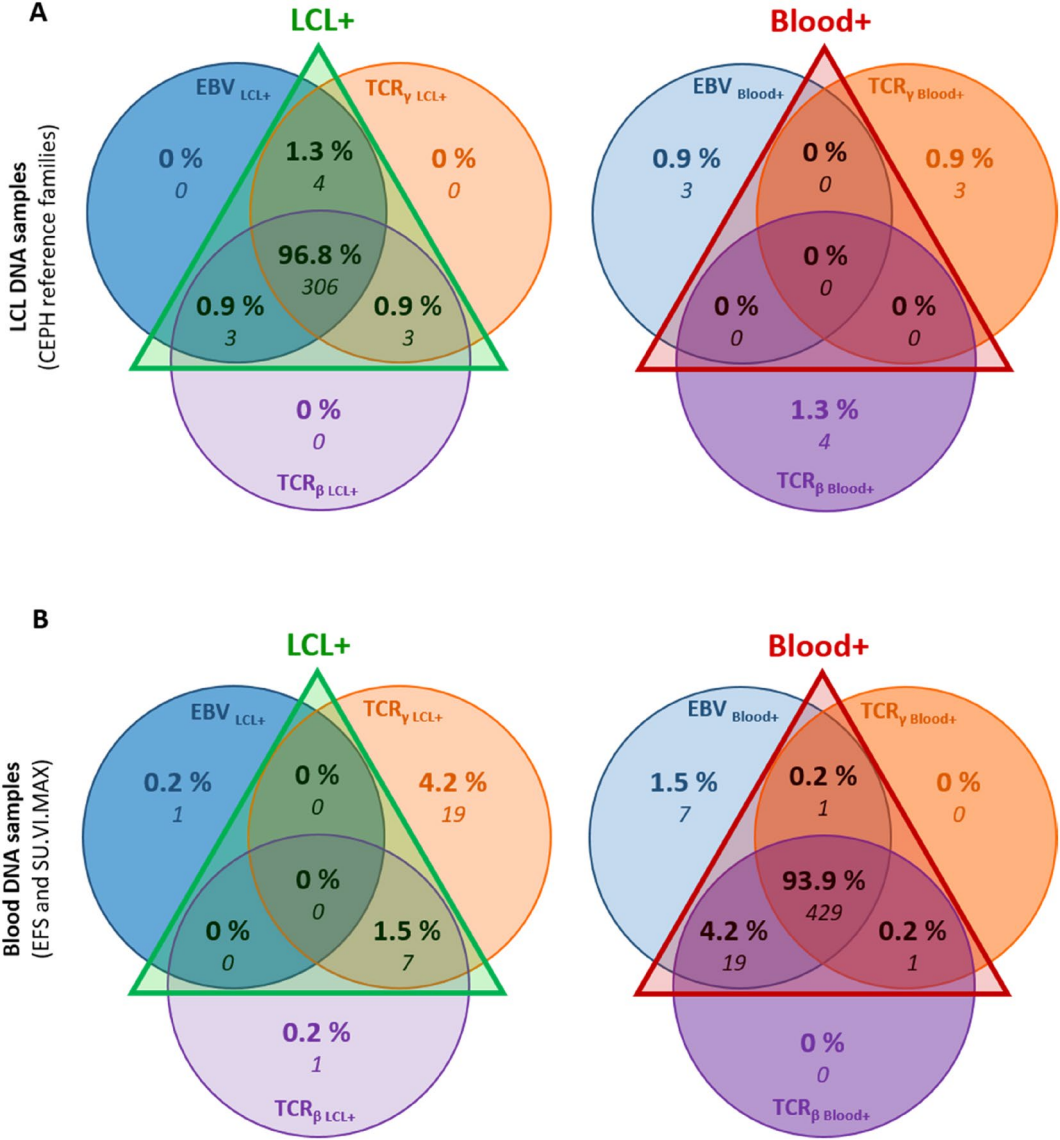
Venn diagrams of the results using combination of the three real-time PCR assays (*EBV*, *TCR*_*γ*_ and *TCR*_*β*_) from blood and LCL DNA of known origin. (**A**) LCL DNA samples from CEPH reference families (n = 316) distribution. (**B**) Blood DNA samples from EFS and SU.VI.MAX (n = 457) distribution. When there was a discrepancy between the results of the three tests, these samples were represented on both the left and right Venn diagrams. For each Venn Diagram, the percentages are calculated from the total number of blood (316 for panel **A**) and LCL (457 for panel **B**) reference DNA samples.

The three tests described above allowed to distinguish blood from LCL DNA samples with high accuracy when used independently (Table 2). However, for further (epi)genomic investigations and applications, we would like to exclude all false positive blood samples (i.e. LCL DNA misclassified as blood DNA) and also to limit the possible technical and/or biological issues that could arise during PCR experiments relying on a single test. We decided to combine our three developed tests and to consider a DNA sample as blood when at least two out of the three tests were positives for blood (Fig. 2 and Table 2). The calculated sensibility (98.5%), specificity (100%), PPV (100%), NPV (97.8%) and accuracy (0.99) showed the best performances compared to the tests used alone equaling the values of *EBV* assay (Table 2). Specificity and PPV calculated using this combination were of particular interest as they indicated no LCL misclassified as blood sample. Thereby, none of the 316 LCL origin samples were false positives (Fig. 2 and Table 2), validating our approach combining the three tests for accurate identification of DNA extracted from blood.

### Application of our tissue-of-origin test to the CEPH Aging cohort

**Figure 3:**
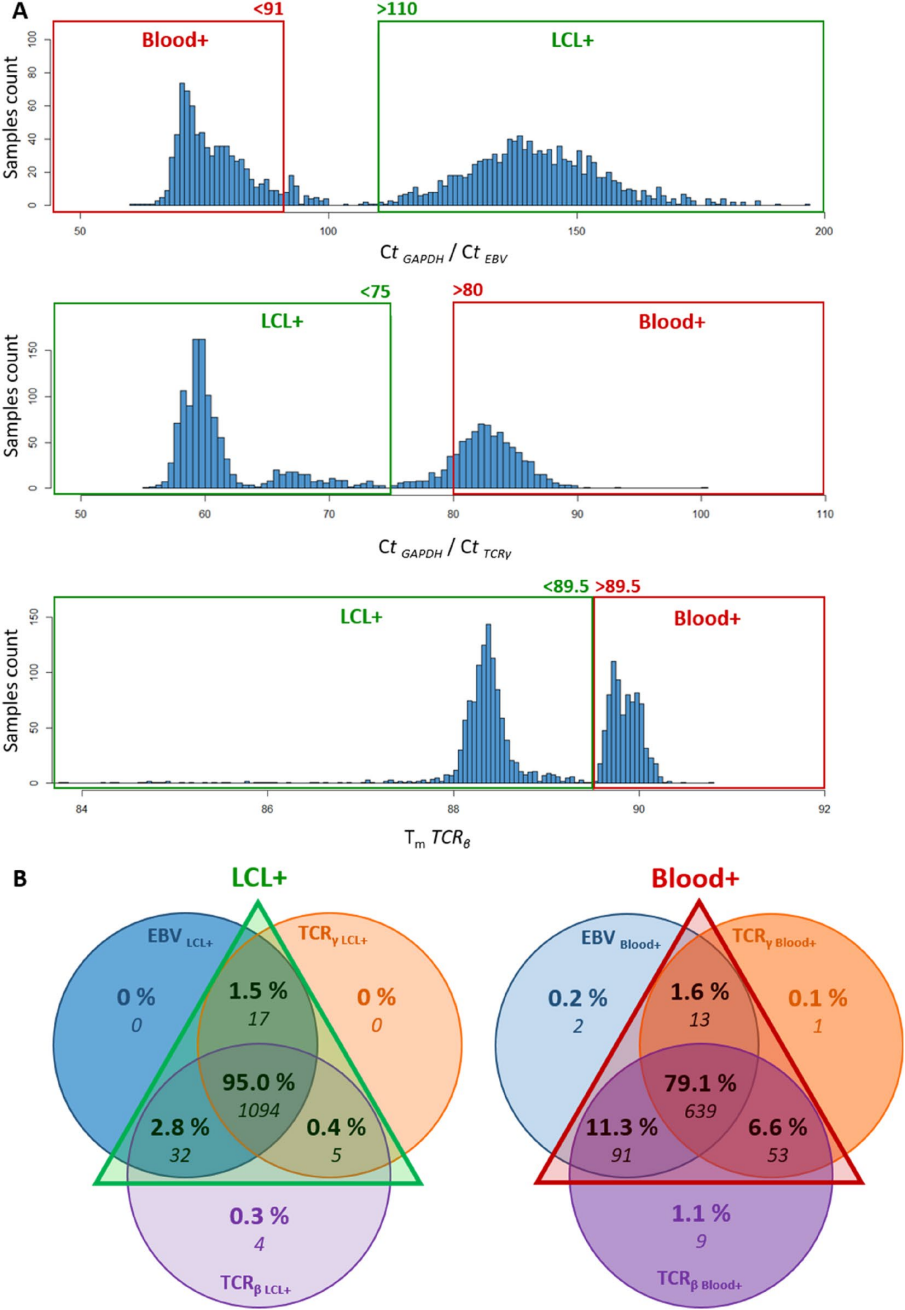
Application of the three tissue-of-origin real-time PCR assays to DNA samples (n = 1957) of the CEPH Aging cohort. (**A**) Distribution of C_t *EBV*_/C_t *GAPDH*_ ratios, C_t *TCRγ*_ /C_t *GAPDH*_ ratios and mean *TCR*_*β*_ T_m_ of CEPH Aging cohort DNA samples based on *EBV*, *TCR*_*γ*_ and *TCR*_*β*_ real-time PCR assays. (**B**) Venn diagrams of the results using the combination of the three real-time PCR assays: *EBV*, *TCR*_*γ*_ and *TCR*_*β*_. When there was a discrepancy between the results of the three tests, the samples were represented on both the left and right Venn diagrams. The percentages were calculated from the total number samples present in each Venn Diagram (1152 for the left and 808 for the right).

Our tissue-of-origin test was applied to 1957 DNA samples from 1813 individuals, including 1346 DNA isolated from nonagenarians and centenarians (NC group) and 457 DNA samples from NC group’s offspring (NCO group) of the CEPH Aging cohort (Table 1). The information about the origin of these DNA samples was dated, incomplete or missing and needed to be validated or identified. The distribution of NC + NCO DNA samples according to C_t *GAPDH*_/C_t *EBV*_ ratio, C_t *GAPDH*_/C_t *TCRγ*_ ratio and *TCR*_*β*_ T_m_ showed the typical bimodal distribution indicating the presence of DNA extracted from blood and LCL in this cohort as expected (Fig. 3A). Using the combination of the three tests, we were able to identify 796 and 1148 DNA samples extracted from blood and LCL, respectively (Fig. 3B and Table 3), while 12 samples remained of uncertain origin despite one blood positive test. When separating NC from NCO DNA samples, our results indicated that the NCO group presented proportionally more DNA samples extracted from blood compared to the NCO group (Supplementary Fig. 3 and 4), probably due to the greater use of DNA samples from the NC group in former genetic studies.

**Table 3:** Concordance between the information present in the CEPH Biobase database and results of real-time PCR tissue-of-origin assays.

Origin of DNA according to the CEPH Biobase database	Tissue-of-origin PCR test			Concordance of DNA origin between database information and PCR test results
	Blood	LCL	Uncertain	
Blood n = 269 (13.74%)	263 (13.44%)	0 (0%)	6 (0.31%)	Blood DNA = 97.77%
LCL n = 1035 (52.89%)	3 (0.15%)	1032 (52.73%)	0 (0%)	LCL DNA = 99.71%
Uncertain n = 653 (33.37%)	530 (27.08%)	116 (5.93%)	7 (0.35%)	Uncertain DNA = 1.07%
Total n = 1957 (100%)	796 (40.67%)	1148 (58.66%)	13 (0.66%)	-

We further compared our results to the information available in the CEPH Biobase database and found 99.31% concordance for the 1304 DNA samples whose tissue of origin information was available (Table 3). Moreover, our combined approach enabled the identification of the tissue-of-origin for 98.93% of the 653 DNA samples whose origin was missing or uncertain according to our database (Table 3). Only 13 out of the 1957 tested DNA samples remained from unknown origin (0.66%, Table 3). Among them, 7 were already uncertain before the test. Taken together, our results allowed to validate the information present in the CEPH Biobase database. They also showed the strength of our high-throughput real-time PCR tissue-of-origin tests applied to a large cohort of DNA samples.

### DNA methylation-based age prediction is altered in lymphoblastoid cell lines

**Figure 4:**
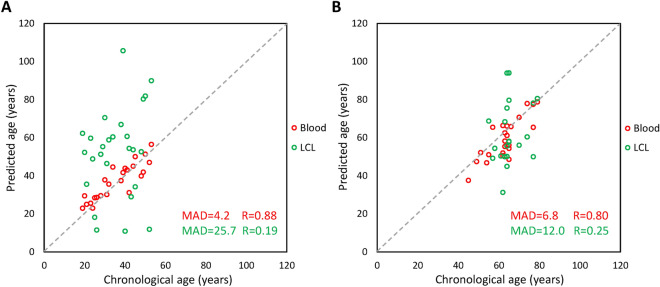
Age predictions of all DNA samples from the DNA methylation-based age prediction model of Thong^44^ using three CpG loci and pyrosequencing. (**A**) Age predictions from EFS blood (n = 24) and CEPH families EBV cell line (n = 26) DNA samples. (**B**) Age predictions from blood (n = 24) and EBV cell line (n = 21) DNA samples of the CEPH Aging cohort. The mean absolute deviation (MAD) of the predicted age from the chronological age and the Pearson R coefficient are given on each graph in red and green for blood and LCL samples, respectively.

The epigenetic clock is defined as the modifications of the epigenomes during aging that correlate to the chronological age similarly in every individual^64^. Thus, several DNA methylation-based age prediction biomarkers have been used to develop age-prediction models principally using pyrosequencing^43–45^ or genome-wide epigenotyping arrays^65–67^. To estimate the age of the samples used in our study and measure the differences of age predictions between blood and LCL DNA, we used the age prediction model of Thong^44^, which is based on DNA methylation of the *KLF14*, *TRIM59* and *ELOVL2* promoters and evaluated as being among the best age prediction models in a previous study^43^. We first evaluated the model on a subset of 24 blood DNA (EFS) and 26 LCL DNA (CEPH families) from control samples of individuals aged from 19 to 53 years (Fig. 4A). The results showed that the age predictions from control blood samples were accurate (MAD = 4.2) and strongly correlated to chronological age (R = 0.88), while the age predictions showed very poor performances for the control EBV samples (MAD = 25.7, R = 0.19, Fig. 4A). Similarly, when the model was applied to 24 blood and 21 LCL DNA samples from nonagenarians and centenarians’ offspring of the CEPH aging cohort aged from 45 to 79 years, the age predictions showed good performances for blood samples (MAD = 6.8, R = 0.80) with a slight tendency for underestimation of the predicted age and poor performances for LCL samples (MAD = 12.0, R = 0.25, Fig. 4B). These results indicated that DNA methylation and the epigenetic clock are impaired in LCL samples and that such analyses should be performed on blood extracted DNA rather than LCL DNA.

## Discussion

The rapid increase in number of genetic and genomic studies in the last thirty years became possible with the development of new high-throughput genotyping and sequencing technologies as well as bioinformatics resources, associated to the reduction of their costs. These studies also required the availability of an ever-growing number of DNA samples that were collected and stored in DNA biobanks or biological resource centers, which also allowed their distribution to the scientific and biomedical community worldwide^68,69^. Thus, several large DNA collections were for the majority established from blood or blood-derived LCLs to provide DNA samples for genetic, genomic and epidemiologic studies^70^. Furthermore, several guidelines, considerations and best practices for biobanking have been proposed aiming to standardize and harmonize the policies and procedures within and between biobanks in order to improve the overall quality and reproducibility of downstream experiments^68,71–73^. Although having being extensively used in genetic, population genetic and genome wide association studies, DNA extracted from LCLs should be used with caution in genomic and more particularly epigenomic studies, as several alterations of their (epi)genomes might arise during immortalization and in vitro culture and might not reflect their cells of origin^28,31–35,38,39^. Thus, the use of genomic DNA extracted from blood should be preferred over LCLs for (epi)genomic studies, and this despite the development of bioinformatics tools that might allow the filtering of LCL-specific alterations before data interpretation^27,28^. In some genomic studies such as the 1000 Genomes Project, whole genome sequencing experiments were performed on DNA samples extracted either from blood or LCLs, and some annotations about the tissue-of-origin could be missing or inaccurate, thereby potentially impacting downstream bioinformatic analyses and the interpretation and significance of the data^39,52^.

In this context, we have developed a rapid and simple high-throughput real-time PCR approach that allowed to distinguish blood extracted from LCL extracted DNA, which was based on the relative detection of EBV genomes and of rearranged *TCR*_*β*_ and *TCR*_*γ*_ (Fig. 1). This tissue-of-origin test is intended to be used as a quality control to validate, ascertain or identify the tissue of origin of DNA samples from large or ancient DNA collections prior to (epi)genomic studies. It could be used at the same time in the sample processing workflow as other quality control tests currently used in DNA biobanks before genotyping or sequencing experiments such as microsatellite markers typing for DNA sample authentication^74^ or sex typing for the detection of potential DNA sample misassignment or mix-up^75^. The use of a *GAPDH* single-copy gene assay was essential to test the amplifiability of DNA and to normalize the *EBV* and *TCR*_*γ*_ assays (Fig. 1 and Supplementary Fig. 1). The three tests could be used independently as they presented good sensitivity and specificity when used alone (Table 2). However, we recommend their use in combination to identify blood DNA samples with a cutoff of two positive tests out of three (Fig. 2, Table 2). Of note, the use of combined tests is considered as an optimal strategy to increase the testing accuracy and reduce the uncertainty compared to single tests^76,77^. Moreover, each individual test could present some drawbacks that should not be shared by the others, thereby justifying the use of three independent tests. For example, the detection of high level of EBV genomes could also be present in DNA extracted from blood from individuals ongoing acute or chronic EBV infection or EBV-associated diseases^53–55,78^, but these health conditions should not impact the results of *TCR*_*β*_ and *TCR*_*γ*_ assays. Although presenting the best individual performances with the control samples, the *GAPDH*/*EBV* assay could also be less sensitive for blood samples from aged individuals with our set cutoff as EBV viral load was known to be higher in the elderly^79,80^, which could potentially explain the moderate shift to the right of the blood extracted DNA sample in our results on the CEPH Aging cohort. This tendency was visible when separating NC from NCO samples, which supported our hypothesis (Fig. 3A and Supplementary Fig. 3A and 4A). When applied to 1957 DNA samples of the CEPH Aging cohort using the thresholds defined with the blood and LCL reference DNA samples, our tissue-of-origin test allowed the identification of 796 DNA extracted from blood and 1148 DNA extracted from LCL, while only 0.66% DNA samples remained of uncertain origin (n = 13, Table 3). These results were compared to the information that was mostly but partially present in the CEPH Biobase database revealing more than 99% agreement on the origin of DNA samples between experimental results and CEPH Biobase information (Table 3). Our tests also allowed the identification of tissue-of-origin for 98.93% DNA samples with missing or uncertain information, enabling their use in downstream (epi)genomic experiments.

Finally, to measure the impact of the origin of our DNA samples on epigenetic analyses, we ran an age prediction model using DNA methylation of three CpG sites on about a hundred individuals from control groups and CEPH Aging collection in order to predict their chronological age (Fig. 4). The age predictions showed good performances for blood DNA (MAD = 4.2–6.8), which were similar to those obtained with DNA methylation-based and pyrosequencing-based age prediction models^43^. Although requiring additional validations, the slight under-estimation of the chronological age observed for the blood DNA samples of the CEPH aging cohort could be of biological and clinical significance (Fig. 4), as the offspring of centenarians was shown to be epigenetically younger and have lower predicted ages^81^. Conversely, age predictions showed very poor performances for LCL DNA (MAD = 12–25.7, Fig. 4). This indicated that the epigenetic clock used was strongly impaired in LCLs and that an age prediction model using as little as three CpG sites could reveal this alteration. Of note, several studies have shown that DNA methylation was altered in LCLs and did not represent the methylome of blood or their cells of origin^31–35^. Few other studies also evaluated age prediction models on LCLs using a high number of CpG sites (> 50) and epigenotyping microarrays data and found the epigenetic clock and age prediction were altered in these cell lines^67,82^. The poorer age prediction performance observed on LCL DNA from CEPH families compared to the CEPH aging cohort might be attributed to the high number of passages for the former, as DNA methylation alterations were described to be stronger in LCLs with high passage numbers^35^. Taken together, our results and the aforementioned studies indicated that when possible, blood extracted DNA should be preferred to LCL DNA for DNA methylation and age prediction analyses.

## Conclusion

Our study presented for the first time an experimental approach for the identification of the tissue of origin of DNA samples, whether extracted from blood or LCLs. It is intended to be used in large and/or ancient DNA collections to validate, ascertain or identify their origin. We proposed this approach as a quality control test that could be implemented in DNA biobanks and used along with other quality control tests prior to (epi)genomic studies. In our experimental conditions, we evaluated the cost per PCR reaction at 1 euro (≈ 1.2 $) for a total of 4 euros (≈ 4.5 $) per DNA sample for the combined approach, which is cost-effective. We also anticipate the development of additional tissue-of-origin tests that could be applied to DNA from other tissue types or from other nucleic acid types, i.e. RNA, which would further improve the practices for biobanks and contribute to the science of biobanking.

## Supplementary Information

Supplementary Information.
