# Supplementary material for: A high-throughput real-time PCR tissue-of-origin test to distinguish blood from lymphoblastoid cell line DNA for (epi)genomic studies
Source: Sci Rep. 2022 Mar 18;12:4684. doi: 10.1038/s41598-022-08663-6 (PMC8933453; doi:10.1038/s41598-022-08663-6)
Supplement: Supplementary file 1 — Supplementary Information. [file 41598_2022_8663_MOESM1_ESM.pdf]

Supplementary information for: A high-throughput real-time PCR tissue-of-origin test to distinguish blood from lymphoblastoid cell line DNA for (epi)genomic studies

Lise M. Hardy[1,2], Yosra Bouyacoub[1,2], Antoine Daunay[1], Mourad Sahbatou[1,3], Laura. G. Baudrin[1,2], Laetitia Gressin[4], Mathilde Touvier[5], Hélène Blanché[4,7], Jean-François Deleuze[1,2,6,7] & Alexandre How-Kit[1,7,ǂ]

[1] Laboratory for Genomics, Foundation Jean Dausset – CEPH, Paris, France

[2] Laboratory of Excellence GenMed, Paris, France

[3] Laboratory for Human Genetics, Foundation Jean Dausset – CEPH, Paris, France

[4] Centre de Ressources Biologiques, CEPH Biobank, Foundation Jean Dausset – CEPH, Paris, France

[5] Sorbonne Paris Nord University, Nutritional Epidemiology Research Team (EREN), Epidemiology and Statistics Research Center Inserm U1153, Inrae U1125, Cnam, University of Paris (CRESS), Bobigny, France

[6] Centre National de Recherche en Génomique Humaine, CEA, Institut François Jacob, Evry, France

[7] Laboratory for Sciences of Biobanking, Paris, France

[ǂ] ***Correspondence to:***

Alexandre How-Kit, Ph.D., Laboratory for Genomics, Foundation Jean Dausset - CEPH, Paris, F-75010, France, Tel.: +33-(0)1- 53725146, email: alexandre.how-kit@fjd-ceph.org

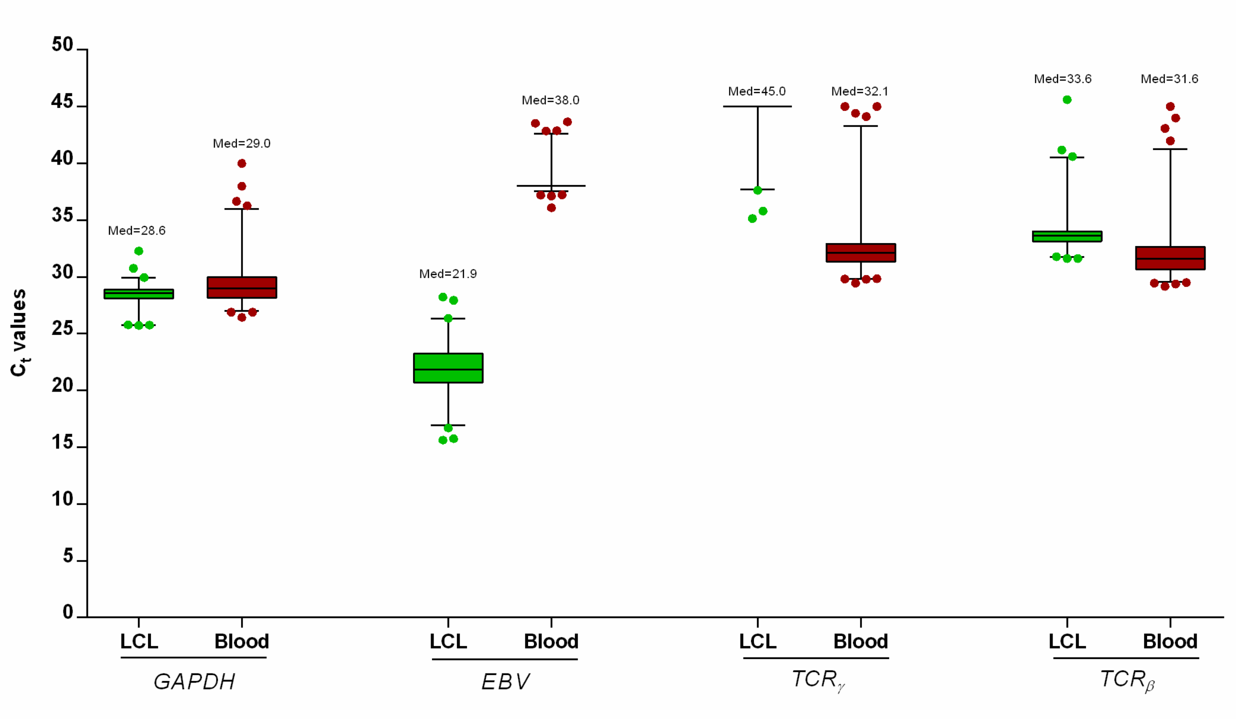

Supplementary figure 1: Raw C_t values of PCR *GAPDH*, *EBV*, *TCR*$_\gamma$ and *TCR*$_\beta$ assays from blood DNA from EFS and SU.VI.MAX (n=457) and LCL DNA from CEPH reference families (n=316). Median values are shown on top of each boxplot.

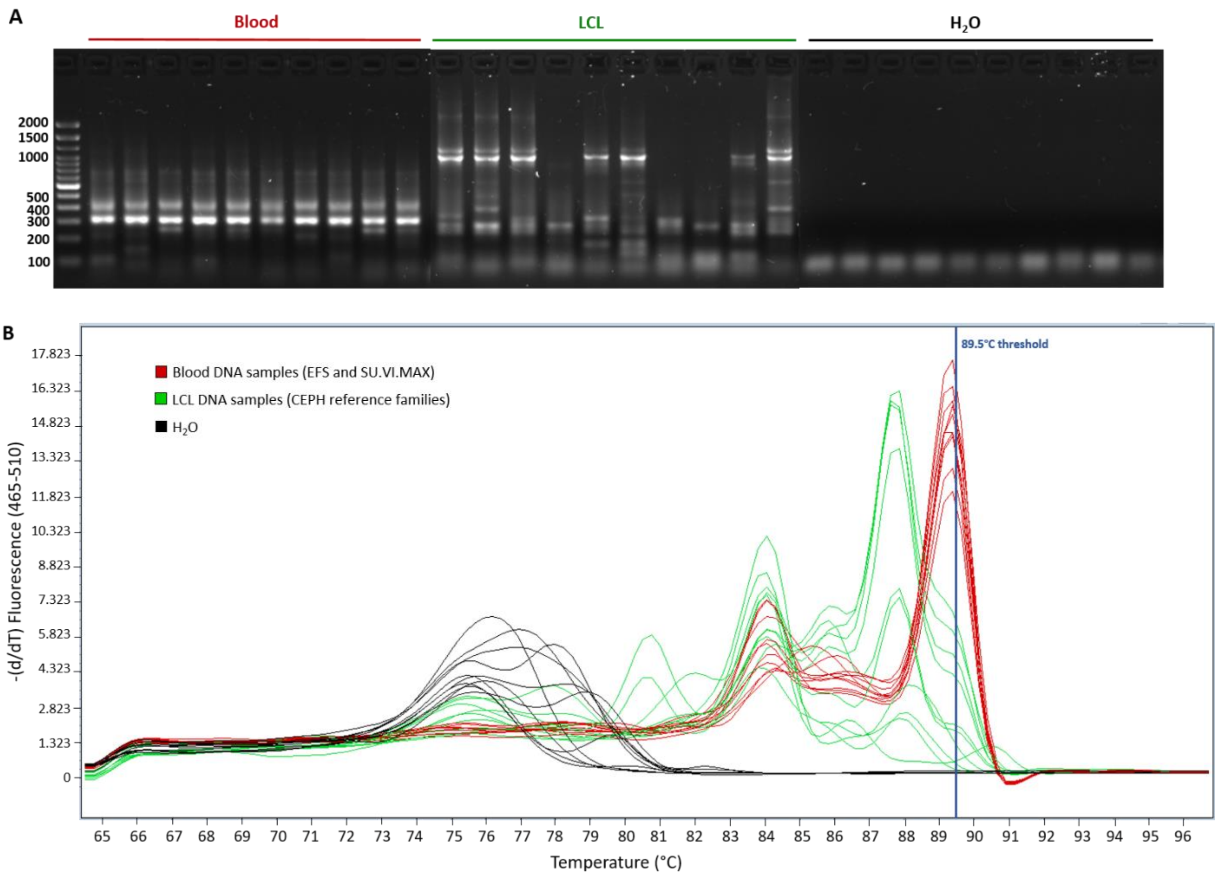

Supplementary figure 2: Characterization of amplification products following TCR_{β} multiplexed PCR (20 µl PCR reactions in a 96-well PCR plate). **A.** Agarose gel electrophoresis of PCR products using blood DNA (n=10), LCL DNA (n=10) and water (n=10) as template. The 300-320 bp PCR bands correspond to specific amplification of rearranged TCR_{β} genes. The panel was made from three different agarose gels at the same scale (blood, LCL and no DNA template). **B.** Melting curve analysis of the same PCR products showing a clear separation between PCR amplicons from blood and LCL DNA samples. Blood DNA are from SU.VI.MAX (n=10) and LCL DNA from CEPH reference families (n=10). The slight T_m shift observed was due to the reaction volume (20 µl) and PCR plate format (96-well).

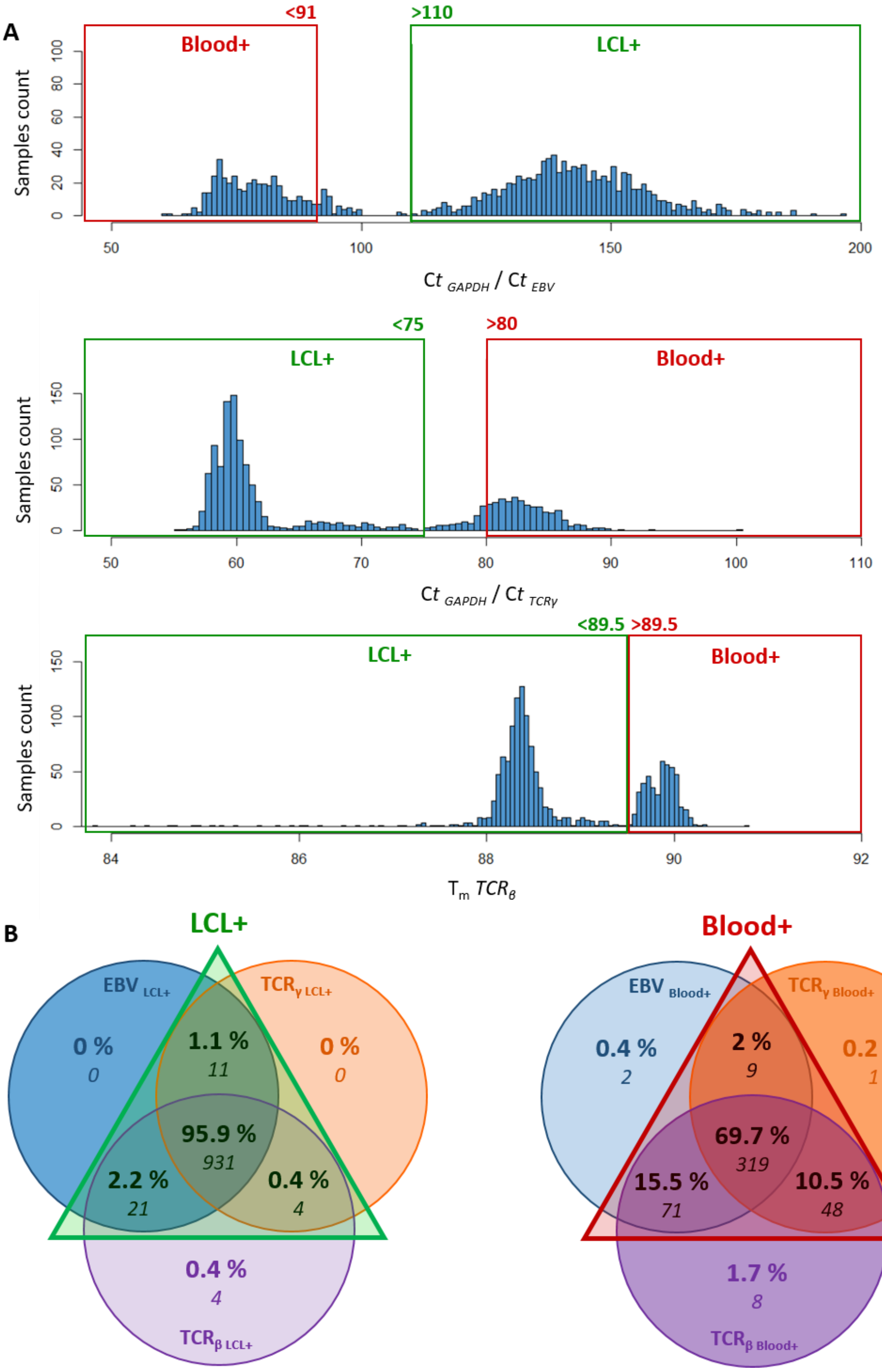

Supplementary figure 3: Application of the three tissue-of-origin real-time PCR assays to DNA samples (n=1957) from the NC group of the CEPH Aging cohort. **A.** Distribution of $C_{t\ EBV}/C_{t\ GAPDH}$ ratios, $C_{t\ TCR\gamma}$ /$C_{t\ GAPDH}$ ratios and mean TCR_β T_m of CEPH Aging cohort DNA samples based on *EBV*, TCR_γ and TCR_β real-time PCR assays. **B.** Venn diagrams of the results using the combination of the three real-time PCR assays: *EBV*, TCR_γ and TCR_β. When there was a discrepancy between the results of the three tests, the samples were represented on both the left and right Venn diagrams. The percentages were calculated from the total number samples present in each Venn Diagram (971 for the left and 458 for the right).

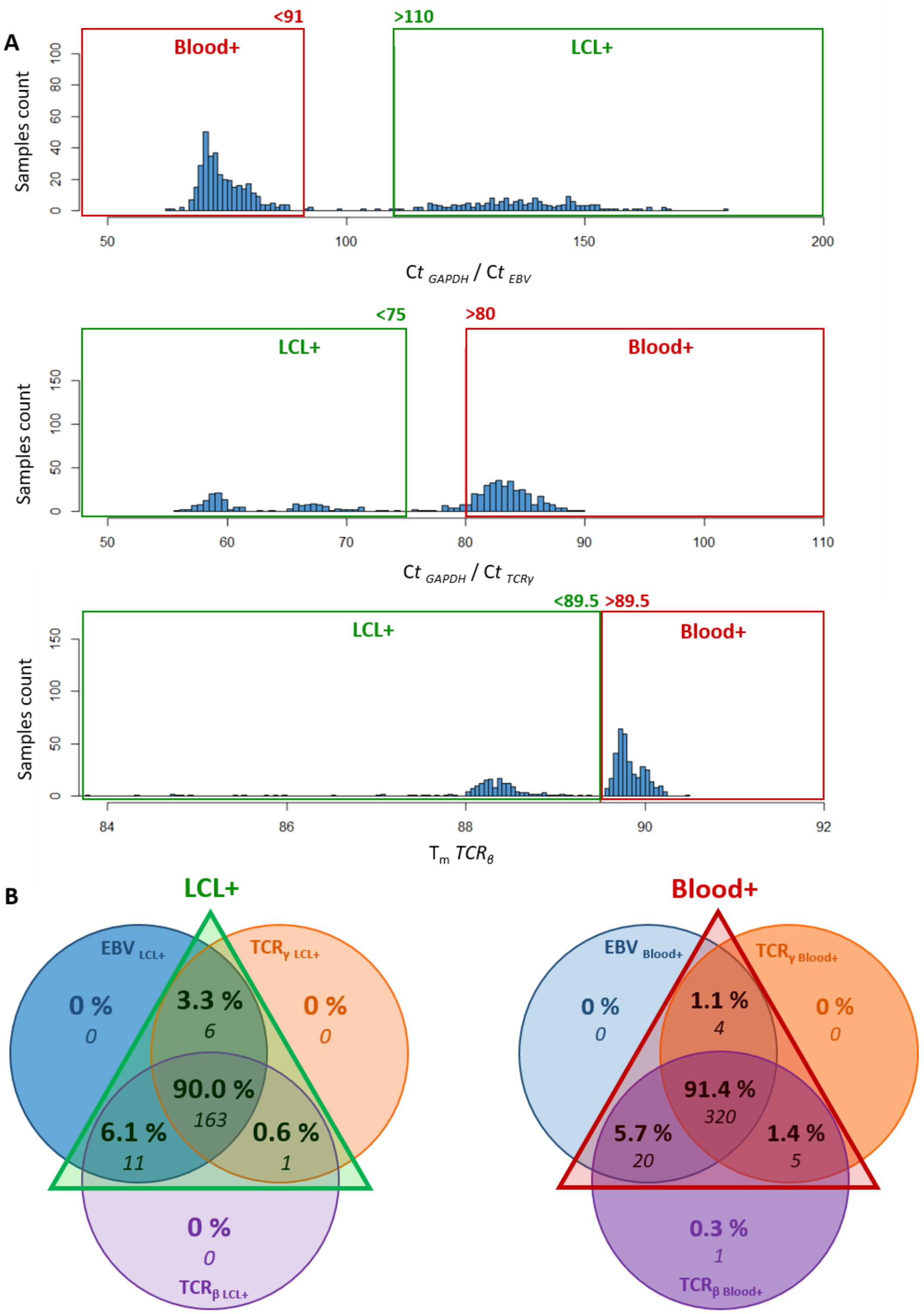

Supplementary figure 4: Application of the three tissue-of-origin real-time PCR assays to DNA samples (n=1957) from the NCO group of the CEPH Aging cohort. **A.** Distribution of $C_{t\ EBV}$/$C_{t\ GAPDH}$ ratios, $C_{t\ TCR\gamma}$ /$C_{t\ GAPDH}$ ratios and mean TCR_{β} T_m of CEPH Aging cohort DNA samples based on *EBV*, TCR_{γ} and TCR_{β} real-time PCR assays. **B.** Venn diagrams of the results using the combination of the three real-time PCR assays: *EBV*, TCR_{γ} and TCR_{β}. When there was a discrepancy between the results of the three tests, the samples were represented on both the left and right Venn diagrams. The percentages were calculated from the total number samples present in each Venn Diagram (181 for the left and 350 for the right).

Supplementary table 1: PCR primer sequences and cycling conditions

Genome/Gene			Sequence	Denaturation		Annealing		Elongation		Amplicon length (pb)	Melting temperature T_m (°C)
				Temp. (°C)	Duration (sec)	Temp. (°C)	Duration (sec)	Temp. (°C)	Duration (sec)		
EBV		F	ACTCGTGCACGTGCTTCTTTAC	95	20	66	20	72	10	170	87
		R	GTGGACTTTGCCAGCCTCTAC								
TCR$_\beta$[1]	D-β-1	F	GCCAAACAGCCTTACAAAGAC	95	30	61	40	72	30	300-320	90
	J-β-1	R	CTTACCTACAACTGTGAATCTGGTG								
	J-β-2	R	CTTACCTACAACGGTTAACCTGGTC								
	J-β-3	R	CTTACCTACAACAGTGAGCCAACTT								
	J-β-4	R	CATACCCAAGACAGAGAGCTGGGTTC								
	J-β-5	R	CTTACCTAGGATGGAGAGTCGAGTC								
	J-β-6	R	CATACCTGTCACAGTGAGCCTG								
TCR$_\gamma$	V-γ-II	F	CTCGAATTCGGAAAGGAATCTGGCATTCCG	95	30	61	30	72	30	190-200	83
	J-γ-1/2	R	CTCGGATCCACCTGTGACAACAAGTGTTGTTC								
GAPDH[2]		F	TCTGCTTCTCTGCTGTAG	95	30	61	30	72	30	100	86
		R	AGGCTGTTGTCATACTTCT								

[1]As *TCR*$_\beta$ is a gene ongoing segment rearrangement, several reverse primers are needed for multiplexed amplifications of a large proportion of rearranged genes.

[2]*GAPDH* is used as a control single-copy gene to normalize C_t data

Supplementary table 2: thresholds used for the three tissue of origin tests based on the results from Figure 1

Genome / Gene	$C_{t\ GAPDH}/C_{t\ Genome/Gene\ of\ interest}$ threshold		T_m threshold (°C)	
	Lower than	Higher than	Lower than	Higher than
EBV	91<br>(Blood+/LCL-)	110<br>(LCL+/Blood-)	-	-
TCRγ	75<br>(LCL+/Blood-)	80<br>(Blood+/LCL-)	-	-
TCRβ	-	-	89.5<br>(LCL+/Blood-)	89.5<br>(Blood+/LCL-)
